# Clinical application of the 2011 IFCPC colposcope terminology

**DOI:** 10.1186/s12905-021-01395-1

**Published:** 2021-06-24

**Authors:** Bei Zhang, Shuhui Hong, Guihui Zhang, Fengnian Rong

**Affiliations:** 1 Department of Obstetrics and Gynecology, Shandong Qianfoshan Hospital, Cheeloo College of Medicine, Shandong University, No.16766, Jingshi Road, Jinan, 250014 Shandong Province China; 2 Department of Pathology, Shandong Qianfoshan Hospital, Cheeloo College of Medicine, Shandong University, Jinan, 250014 Shandong China

**Keywords:** 2011 IFCPC terminology, Colposcopy, Minor changes, Major changes, Lugol’s staining

## Abstract

**Background:**

Colposcopy offers an accurate way to the diagnose of cervical precancerous lesions. However, the diagnostic accuracy of colposcopy is unsatisfied. This study was to evaluate colposcopic accuracy according to the 2011 International Federation of Cervical Pathology and Colposcopy (IFCPC) terminology.

**Methods:**

A retrospective cohort study was performed in 1,838 patients who underwent colposcopy in Shandong Qianfoshan Hospital, Cheeloo College of Medicine, Shandong University from October 2013 to April 2018. Using conization or cervical biopsy pathology as the gold standard, the agreement between colposcopic diagnosis and pathologic diagnosis was calculated, and correlations between variables were analyzed.

**Results:**

As an authoritative and widely used terminology for colposcopy diagnosis, the 2011 IFCPC terminology has certain clinical practicality and diagnostic accuracy. However, some signs such as mosaic, punctation, sharp border, inner border sign and ridge sign had high specificity but unsatisfactory sensitivity, which limited the diagnostic value. Therefore, we discussed the Lugol’s staining, a very common sign in colposcopy, and analyzed the diagnostic significance of bright yellow staining in low-grade squamous intraepithelial lesion (LSIL) and mustard yellow staining in high-grade squamous intraepithelial lesion (HSIL). The results showed that mustard yellow may be a valuable indicator in the diagnosis of HSIL.

**Conclusion:**

The 2011 IFCPC colposcope terminology has standardized interpretations of the colposcopic findings and improved the accuracy of colposcopy diagnosis. The aceto-white epithelium still has important diagnostic value; however, the value of a few signs is needed to be discussed and new signs are expected to be discovered. Although the significance of Lugol’s staining was diminishing, mustard yellow might be a valuable indicator for the diagnosis of HSIL.

## Background

Results released by the International Agency for Research on Cancer show that in 2018, there were an estimated 570,000 new cases of cervical cancer worldwide and 310,000 deaths from cervical cancer. Among them, nearly 110,000 new cases of cervical cancer and nearly 50,000 deaths occurred in China [1]. However, persistent infection with high-risk human papilloma virus (hrHPV) appears to be the major driver of cervical cancer development. It is a long process from precancerous lesions initiated by HPV infection to cervical cancer, so the diagnosis and treatment of precancerous lesions are particularly important. With the role of identifying the lesion, guiding biopsy and helping to plan treatment and follow-up, colposcopy, in conjunction with cervical screening has played an important role in reducing the incidence of cervical cancer. However, colposcopy is considered as subjective procedure which is highly dependent on the knowledge and skill of the observer [2–5]. Therefore, standardizing the colposcopy evaluation has always been the subject of concern and discussion. The Reid Colposcopic Index (RCI), the modified RCI, and the Swede score have all been used historically in colposcopic diagnosis. Although there are various colposcopy scoring systems, there is no consensus on the standardization [6–9]. The International Federation of Cervical Pathology and Colposcopy (IFCPC), which is the current authoritative international organization of cervical pathology and colposcopy, has presented four versions of colposcopic terminology in 1975, 1990, 2002, and 2011 with the purpose of promoting uniform colposcopy terminology and practice. The American Society for Colposcopy and Cervical Pathology (ASCCP) proposed ASCCP Colposcopy Standards in 2017 based on colposcopy practice in the United States. On the one hand, all the colposcopy terminology changes reflect the continuous development of colposcopy technology in recent years and the improvement in our understanding of colposcopy; on the other hand, there are no accurate colposcopy standards that have been widely accepted and applied worldwide. Therefore, colposcopy standards will continue to evolve moving forward. In this study, we discuss the advantages and disadvantages of the 2011 IFCPC colposcopy terminology in clinical applications.

## Methods

### Subjects and procedures

A retrospective study of 1,838 patients with abnormal cervical cytology (atypical squamous cells of uncertain significance; atypical squamous cell not exclude high-grade squamous intraepithelial lesion; low-grade squamous intraepithelial lesion; high-grade squamous intraepithelial lesion; atypical glandular cells; and invasive cervical cancer), positive high-risk HPV testing, symptoms of contact bleeding, vaginal discharge, or suspicious-looking cervixes was carried out. All patients underwent colposcopy in Shangdong Qianfoshan Hospital, Cheeloo College of Medicine, Shandong University from October 2013 to April 2018. The women who had hysterectomy or history of pelvic radiation, who underwent colposcopy but had no histopathologic diagnosis and complete data, who had immunosuppressive diseases were excluded from this study. All selected cases had a pathological diagnosis based on a cervical biopsy or a cervical cone resection. 2–4 targeted biopsies were taken from the abnormal areas. If the colposcopy did not reveal any lesions, but it was unsatisfactory, a four-quadrant biopsy from the squamous column junction and endocervical curettage were taken. Biopsy was not performed when the colposcopy was satisfactory and did not reveal any lesions. These cases were not included in this analysis. Mean patient age was 41.7 years (41.7 ± 10.6 years). Leisegang BG/LED Y/C optoelectronic integrated digital colposcope was used, and images were obtained using a Canon EOS600D camera. Patients received colposcopic diagnoses according to the 2011 IFCPC colposcopic terminology by two colposcopists with 5–7 years working experience in colposcopy. Routine colposcopy was performed, which involved a general view of the cervix without reagent, a 3% acetic acid test, and a 5% Lugol’s iodine staining test. The study was conducted in accordance with the principles of the Declaration of Helsinki and received ethical approval from the Medical Ethics Committee of Shandong Provincial Qianfoshan Hospital, Shandong University(2020S554). Because of the retrospective nature of the study, the Medical Ethics Committee of Shandong Provincial Qianfoshan Hospital, Shandong University approved there was no need for consent to participate to be obtained.

### 2011 IFCPC colposcopic diagnosis

The 2011 IFCPC terminology was applied to the colposcopy image description and diagnosis as follows [10]: (1) General assessment: adequate or inadequate; squamocolumnar junction visibility: completely, partially, not visible; transformation zone types (TZ) 1, 2, 3; (2) Normal colposcopic findings: original squamous epithelium; columnar epithelium; ectopy; metaplastic squamous epithelium; nabothian cysts; crypt openings; (3) Abnormal colposcopic findings: general principles (location of the lesion, size of the lesion and size of the lesion as percentage of cervix); Grade 1 (minor): thin acetowhite epithelium, fine mosaic, fine punctation, irregular, geographic border; Grade 2 (major): dense acetowhite epithelium, coarse mosaic, coarse punctuation, sharp border, inner border sign, ridge sign, rapid appearance of acetowhitening, cuffed crypt openings; nonspecific: leukoplakia, erosion, Lugol’s staining; suspicious for invasion: atypical vessels, necrosis, ulceration, tumor or gross neoplasm; miscellaneous findings included: condyloma, polyp, inflammation, stenosis, endometriosis; 4) Colposcopic diagnoses were classified as normal or benign, low-grade squamous intraepithelial lesion (LSIL), high-grade squamous intraepithelial lesion (HSIL) and invasive carcinoma.

### Pathological diagnosis

Pathological diagnosis was divided into [11]: normal or benign, LSIL, HSIL, and carcinoma according to the 2012 Lower Anogenital Squamous Terminology. LSIL included cervical intraepithelial neoplasia (CIN)1, or P16 negative CIN2, koilocytosis, flat condyloma; HSIL included CIN3 or P16 positive CIN2. Cervical biopsy diagnosis was used as the pathological diagnosis for patients without conization, while the final histopathologic diagnosis was applied to those who underwent conization or a hysterectomy.

### Statistical methods

The estimated agreement between colposcopic and histological diagnoses was determined using weighted kappa statistics. The association between lesion size and pathological diagnosis was conducted using the Mantel–Haenszel χ^2^ test. Sensitivity, specificity, positive predictive value (PPV), negative predictive value (NPV), Youden’s index (YI), and their 95% confidence intervals (CIs) were used to assess accuracy. The area under the curve (AUC) was used in logistics analysis to compare the diagnostic value of bright yellow for LSIL and mustard yellow for HSIL. Data analysis was performed using SAS 9.4, while the Delong test was used to compare receiver operating characteristic (ROC) curves using MedCalc statistical software. A two-sided *P* < 0.05 was set as being statistically significant.

## Results

### Agreement between colposcopic and histological diagnoses

Data from 1,838 patients were analyzed in this study. The colposcopic diagnosis and histological diagnosis were consistent in 1,196 cases (65.1%) with a weighted kappa = 0.60 (95% CI = 0.57–0.63, *P* < 0.001). Agreement between colposcopic diagnosis and cervical histopathology is shown in Table 1. In cases diagnosed as normal or benign, LSIL, HSIL, or carcinoma, the agreement between the colposcopic diagnosis and the histological diagnosis were 43.2% (252/583), 75.3% (482/640), 74.2% (385/519), and 80.2% (77/96), respectively. Of 1,838 cases, 22.0% of patients (404/1838) were overdiagnosed using colposcopy while 13.0% (238/1838) were underdiagnosed. 307 of 404 patients (76.0%) histologically diagnosed as normal or benign were overdiagnosed as having LSILs using colposcopy. 116 of 238 patients (48.7%) histologically diagnosed as having HSILs were underdiagnosed as having LSILs using colposcopy. 93 of 238 patients (39.1%) histologically diagnosed as having LSILs were underdiagnosed as normal or benign using colposcopy.

**Table 1:** Agreement between colposcopic diagnosis and cervical histopathology

Colposcopic diagnosis	Histopathological diagnosis				
	Normal or benign	LSIL	HSIL	Carcinoma	Total
Normal or benign	252	93	10	2	357
LSIL	307	482	116	2	907
HSIL	24	65	385	15	489
Carcinoma	0	0	8	77	85
Total	583	640	519	96	1838

LSIL, low-grade squamous intraepithelial lesion; HSIL, high-grade squamous intraepithelial lesion;

The sensitivity, specificity, PPV, NPV and YI of colposcopic diagnosis for normal cervix from any cervical lesion (LSIL, HSIL and carcinoma) and HSIL^+^ (HSIL and carcinoma) form LSIL^−^ (LSIL/normal or benign) were 43.2%, 91.6%, 70.6%, 77.7%, 0.349 and 92.7%, 78.9%, 89.7%, 84.5%, 0.716 respectively. The accuracy of colposcopic diagnoses in distinguishing cervical histopathology when HSIL and LSIL respectively as the cutoffs is shown in Table 2. When taking LSIL as the cutoff, the specificities was improved, the sensitivities was decreased, and the YI was not as good as those when HSIL was used as the cutoff.

**Table 2:** Accuracy of colposcopic diagnoses in distinguishing cervical histopathology at different cutoffs

	Sensitivity (95% CI)	Specificity (95% CI)	Positive predictive value (95% CI)	Negative predictive value (95% CI)	Youden index
LSIL/HSIL/carcinoma vs. normal or benign	43.2% (39.3–47.3%)	91.6% (90.0–93.1%)	70.6% (65.7–75.1%)	77.7% (75.5–79.7%)	0.349
HSIL/carcinoma vs. LSIL/normal or benign	92.7% (91.1–94.1%)	78.9% (75.5–81.9%)	89.7% (87.9–91.3%)	84.5% (81.3–87.2%)	0.716

LSIL, low-grade squamous intraepithelial lesion; HSIL, high-grade squamous intraepithelial lesion

### General assessment of colposcopic findings

Of the 1,838 patients, 1,691 (1691/1838, 92.0%) had adequate colposcopic assessment and 147 (147/1838, 8.0%) had inadequate colposcopic assessment. The squamocolumnar junction was completely, partially and not visible in 334 (334/1838, 18.2%), 745 (745/1838, 40.5%) and 759 (759/1838, 41.3%) cases respectively.

Transformation zone types 1, 2, 3 accounted for 16.8% (309/1838), 1.4% (25/1838) and 81.8% (1504/1838).

### Abnormal colposcopic findings

Of the 1,838 patients, 1,675 had abnormal colposcopic findings on < 30% of the visible cervix (91.1%, 1675/1838), 121 had abnormal findings on 30–67% of the visible cervix (6.6%, 121/1838), and 42 on > 67% of the visible cervix (2.3%, 42/1838). The linear trend test of lesion size and pathological diagnosis showed the larger the size of the lesion, the more serious the disease (χ^2^ = 261.869, *P* < 0.001).

The thin acetowhite epithelium, fine punctation and fine mosaic were regarded as minor changes for LSIL. In grade 1, the sensitivity, specificity, PPV, NPV and YI of detecting LSIL were 87.0%, 59.0%, 53.2%, 89.5% and 0.461. Those of the fine mosaic and fine punctation are shown in Table 3. In grade 2, the sensitivity, specificity, PPV, NPV and YI of thick white acetate epithelium in the diagnosis of HSIL were 73.2%, 87.9%, 70.4%, 89.3% and 0.611. Other data are shown in the Table 4. It was generally believed that thick white acetate epithelium had high diagnostic value. The specificity of coarse mosaic, coarse punctuation, cuffed crypt openings, sharp border, inner border sign and ridge sign were all higher than 90%. Although rare, inner border and ridge sign—two new colposcopic signs had high PPV of 84.6% and 80.0% respectively, prior to the other signs including thick white acetate epithelium.

**Table 3:** Accuracy of colposcopic minor changes in predicting low-grade lesion

	Sensitivity (95% CI)	Specificity (95% CI)	Positive predictive value (95% CI)	Negative predictive value (95% CI)	Youden index
Thin aceto-white epithelium	87.0% (84.2–89.4%)	59.0% (56.2–61.8%)	53.2% (50.1–56.2%)	89.5 (87.2–91.5%)	0.461
Fine mosaic	0.6% (0.2–1.7%)	99.4% (98.8–99.7%)	36.4% (15.0–64.8%)	65.2% (63.0–67.3%)	0.045
Fine punctation	14.5% (12.0–17.5%)	89.3% (87.4–91.0%)	42.1% (35.8–48.7%)	66.2% (63.8–68.4%)	0.035

**Table 4:** Accuracy of colposcopic major changes in predicting high-grade lesion

	Sensitivity (95% CI)	Specificity (95% CI)	Positive predictive value (95% CI)	Negative predictive value (95% CI)	Youden index
Dense aceto-white epithelium	73.2% (69.2–76.9%)	87.9% (86.0–89.6%)	70.4% (66.4–74.1%)	89.3% (87.5–90.9%)	0.611
Coarse mosaic	17.0% (14.0–20.4%)	97.6% (96.6–98.3%)	73.3% (64.8–80.5%)	74.9% (72.8–76.9%)	0.145
Coarse punctation	28.5% (24.8–32.6%)	94.9% (93.6–96.0%)	68.8% (62.4–74.7%)	77.1% (75.0–79.1%)	0.234
Sharp border	4.8% (3.3–7.0%)	99.1% (98.0–99.5%)	67.6% (51.4–80.5%)	72.6% (70.5–74.6%)	0.039
Inner border sign	2.1% (1.1–3.8%)	99.9% (99.4–100.0%)	84.6% (56.5–96.9%)	72.2% (70.1–74.2%)	0.020
Ridge sign	2.3% (1.3–4.0%)	99.8% (99.3–100.0%)	80.0% (54.1–93.7%)	72.2% (70.1–74.2%)	0.021
Cuffed crypt openings	35.45% (31.5–39.7%)	91.1% (89.5–92.6%)	61.1% (55.5–66.5%)	78.2% (76.1–80.2%)	0.266

Because of several studies showed poor reliability of Lugol’s staining, it was removed from the “minor grade” category to the “nonspecific” category [7, 12, 13]. In this study, Lugol’s staining negativity had a high sensitivity and NPV while the specificity was low. The data are shown in the Table 5. But compared with rare sharp border, inner border sign, ridge sign and even mosaic, punctuation, Lugol’s staining negativity was very common.

**Table 5:** Accuracy of colposcopic Lugol’s staining negativity in predicting normal or benign, low-grade lesion, high-grade lesion and carcinoma

	Sensitivity (95% CI)	Specificity (95% CI)	Positive predictive value (95% CI)	Negative predictive value (95% CI)	Youden index
Normal or benign	81.3% (77.9–84.3%)	1.5% (1.0–2.4%)	27.7% (25.7–29.9%)	14.8% (9.6–22.1%)	− 0.172
LSIL	98.0% (96.5–98.8%)	9.6% (8.1–11.4%)	36.7% (34.4–39.0%)	89.8% (83.3–94.1%)	0.076
HSIL	99.0% (97.7–99.6%)	9.3% (7.9–11.0%)	30.1% (27.9–32.3%)	96.1% (90.9–98.6%)	0.084
Carcinoma	99.0% (93.8–100.0%)	7.3% (6.2–8.6%)	5.6% (4.6–6.8%)	99.2% (95.3–100.0%)	0.062

LSIL, low-grade squamous intraepithelial lesion; HSIL, high-grade squamous intraepithelial

According to the degree of yellow, the Lugol’s staining negativity was divided into bright and mustard yellow. The sensitivity and NPV of bright yellow to LSIL diagnosis were higher than fine mosaic and fine punctation, only lower than thin acetowhite epithelium which is shown in Table 6. Mustard yellow had high specificity and NPV of HSIL diagnosis. The YI was lower than dense aceto-white epithelium but higher than coarse mosaic, coarse punctuation, cuffed crypt openings, sharp border, inner border sign and ridge sign. The data is shown in Table 7. The accuracy of colposcopic Lugol’s staining bright yellow in predicting LSIL^−^ (LSIL and normal or benign) and Lugol’s staining mustard yellow in predicting HSIL^+^ (HSIL and carcinoma) is shown in Table 8. We performed further statistical analysis on the diagnostic value of Lugol’s staining. According to the construction of the logistics regression model, the AUC under the ROC curve evaluation model was to predict the values of bright yellow and mustard yellow in the diagnosis of LSIL and HSIL. The results showed that the odds ratio (OR) of bright yellow for LSIL was 1.11 (95% CI 0.86–1.44, *P* = 0.411). In predicting LSIL, thin acetowhite epithelium, fine mosaic, fine punctation with and without the inclusion of bright yellow, the diagnostic efficacy AUC of the model increased from 0.738 (0.717–0.758) to 0.741 (0.720–0.761) (*P* > 0.05). The odds ratio (OR) of mustard yellow for HSIL was 1.43 (95% CI 1.06–1.94, *P* = 0.019). In predicting HSIL, dense acetowhite epithelium, coarse mosaic, coarse punctuation, sharp border, inner border sign, ridge sign, cuffed crypt openings with and without the inclusion of mustard yellow, the diagnostic efficacy AUC of the model increased from 0.835 (0.817–0.852) to 0.839 (0.822–0.856) (*P* > 0.05). As a result, mustard yellow might be a valuable indicator for the diagnosis of HSIL.

**Table 6:** Accuracy of colposcopic Lugol’s staining bright yellow in predicting normal or benign, low-grade lesion, high-grade lesion and carcinoma

	Sensitivity (95% CI)	Specificity (95% CI)	Positive predictive value (95% CI)	Negative predictive value (95% CI)	Youden index
Normal or benign	70.3% (66.5–73.9%)	35.8% (33.2–38.5%)	33.7% (31.1–36.4%)	72.2% (68.5–75.6%)	0.061
LSIL	75.6% (72.2–78.8%)	38.9% (36.2–41.7%)	39.8% (37.1–42.6%)	74.9% (71.4–78.2%)	0.145
HSIL	49.9% (45.6–54.2%)	27.5% (25.1–29.9%)	21.3% (19.1–23.7%)	58.2% (54.3–62.0%)	− 0.227
Carcinoma	65.6% (55.7–74.4%)	33.8% (31.6–36.1%)	5.2% (4.1–6.6%)	94.7% (92.6–96.2%)	0.062

LSIL, low-grade squamous intraepithelial lesion; HSIL, high-grade squamous intraepithelial lesion

**Table 7:** Accuracy of colposcopic Lugol’s staining mustard yellow in predicting normal or benign, low-grade lesion, high-grade lesion and carcinoma

	Sensitivity (95% CI)	Specificity (95% CI)	Positive predictive value (95% CI)	Negative predictive value (95% CI)	Youden index
Normal or benign	11.0% (8.7–13.8%)	65.7% (63.1–68.3%)	13.0% (10.3–16.2%)	61.4% (58.8–64.0%)	0.061
LSIL	22.3% (19.3–25.7%)	70.7% (68.1–73.2%)	29.0% (25.1–33.1%)	63.0% (60.4–65.6%)	− 0.070
HSIL	49.1% (44.9–53.4%)	81.9% (79.7–83.9%)	51.6% (47.2–56.0%)	80.4% (78.2–82.4%)	0.310
Carcinoma	33.3% (24.7–43.3%)	73.5% (71.4–75.5%)	6.5% (4.6–9.0%)	95.2% (93.5–96.5%)	0.068

LSIL, low-grade squamous intraepithelial lesion; HSIL, high-grade squamous intraepithelial lesion;

**Table 8:** Accuracy of colposcopic Lugol’s staining bright yellow in predicting LSIL^−^ (LSIL and normal or benign) and Lugol’s staining mustard yellow in predicting HSIL^+^ (HSIL and carcinoma)

	Sensitivity (95% CI)	Specificity (95% CI)	Positive predictive value (95% CI)	Negative predictive value (95% CI)	Youden index
Lugol’s staining bright yellow/LSIL^−^	73.1% (70.5–75.5%)	47.6% (43.7–51.6%)	73.5% (71.0–75.92%)	47.1% (43.2–51.0%)	0.207
Lugol’s staining mustard/HSIL^+^	46.7% (42.8–50.6%)	83.1% (80.9–85.1%)	58.1% (53.7–62.4%)	75.6% (73.2–77.8%)	0.297

LSIL, low-grade squamous intraepithelial lesion; HSIL, high-grade squamous intraepithelial lesion

LSIL^−^ (LSIL and normal or benign); HSIL^+^ (HSIL and carcinoma)

Invasive carcinoma was rare. Atypical vessels were classified as suspicious for invasion and it had certain diagnostic value for microinvasive carcinoma. Miscellaneous findings included condyloma, polyp, inflammation, stenosis and endometriosis. Among 25 patients with condyloma, normal or benign was found in only 1 patient, LSIL in 17 and HSIL in 7 patients. Among 94 patients with polyp, normal or benign was found in 52 patients, LSIL in 26, HSIL in 15 and carcinoma in 1 patient. In those with endometriosis, most (8/11) were diagnosed with inflammation.

## Discussion

With the purpose of unifying the nomenclature of colposcopy for comparative studies and improving the accuracy of diagnosis, IFCPC presented its first International Colposcopic Classification in 1975, its second nomenclature in 1990 and third in 2002. In 2011, the IFCPC committee examined the past IFCPC terminologies and proposed an evidence-based terminology by reviewing publications. It was recommended that the 2011 terminology should replace all other terminologies and be implemented immediately for diagnosis, treatment and research [14]. So far, the 2011 IFCPC terminology has been proposed for several years. It has certain clinical practicability. Several studies demonstrated it could improve the colposcopic accuracy. However, the reproducibility of transformation zone and the predictive value of a few signs remained to be questioned. Meanwhile, with the popularized of HPV vaccine and changes in cervical cancer screening strategies, colposcopy presents new challenges.

In this study, we analyzed the clinical applicability of the 2011 IFCPC nomenclature in predicting cervical disease. The results showed the agreement between histopathology and colposcopy was 65.1% with weighted kappa = 0.597. It was equal to Li et al.’s of 64.95% with consistency of kappa = 0.436, Fan et al.’s of 65.5% with weighted kappa strength 0.494 and Prabhakaran’s of 65.7% [15–17]. Although IFCPC nomenclature was only moderate, it was better than Swede Score, RCI, modified RCI and 2002 IFCPC nomenclature [7, 8, 18–22]. In our study, we found that the 2011 IFCPC colposcopic terminology had a high sensitivity (92.7%) in differentiating HSIL^+^ from LSIL^−^, higher than that reported in previous studies (30–91.3%) [23]. The specificity for detecting HSIL^+^ was 78.9%, a little lower than previously reported (79–96.5%) [21, 24, 25]. The PPV and NPV of colposcopy to diagnose HSIL^+^ were 89.7% and 84.5%, both comparable to the previous findings [21, 23–25]. The term of cervical colposcopy in 2011 begins with “general assessment” with the purpose of emphasizing the level of reliability of this colposcopic examination [14]. In our study, 1.3% (147/1838) of all patients had inadequate colposcopic examination. The main reason was bleeding, others included scarring of lacerations, vaginal wall relaxation, changes in cervical position (myoma compression, adhesion), inflammation and neoplasm. This reminds us colposcopic operation should be gentle, so as not to artificially cause contact bleeding, especially near the endocervical canal. For changes in cervical position, we can use tools such as cervical clamp when necessary to help fully exposing the cervical transformation zone. If there is cervical neoplasm, it should be pushed in different directions in order to assess the transformation zone at 360°. The squamocolumnar junction was completely visible in 334 (334/1838, 18.2%). “Partially visible” and “not visible” are respectively defined as mostly visible and most or all invisible of the squamocolumnar junction because it is in the endocervical canal. We think the definitions of “partially visible” and “not visible” are ambiguous. It is difficult to grasp the degree of “most squamous column junctions visible and invisible”. We suggest the visibility of squamocolumnar junction in the range of 0°–360° is defined as “partially visible” with visible rang indicated as necessary. For example, the squamocolumnar junction is partially visible from 90° to 180°. It is also suggested “not visible” means the squamous column junction is completely invisible.

Once the highlight but now the controversy of 2011 IFCPC nomenclature is cervical TZ. The authorsʼ supposition of TZ is that it advances a closer relationship to therapeutic strategies and leads to individualized treatment [26]. English guidelines recommend adjusting loop length to correspond to TZ type. However, in clinical practice of several years, the reproducibility of TZ in different examiners has been questioned. In this study, transformation zone types 1, 2, 3 accounted for 16.8% (309/1838), 1.4% (25/1838) and 81.8% (1504/1838). Li et al.’s study of 525 cases indicated types 1, 2, and 3 of TZs accounted for 22.3%, 7.2%, and 70.5% [15]. Fan et al.’s research showed 1005 cases (44.4%, 1005/2262) were classified as type 2 TZ, 887 (39.2%, 887/2262) as type 1 and 370 (16.4%, 370/2262) as type 3 TZ [16]. It was significantly different between the distributions of the three types TZ in our and Fan et al.’s studies, especially of type 2 TZ. In the German analysis of 3761 patients, 2153 cases (57%) were classified as type 2 TZ, 906 cases (24%) were type 1 TZ, 702 cases (19%) were type 3 TZ, and significant heterogeneity of TZs in different clinics was showed [27]. In 2017, ASCCP claimed that literature suggested the use of TZ type unrepeatable, especially for type 2 TZ, and there was no evidence showed TZ type can improve the prediction or management of cervical disease [22, 27]. Therefore, TZ types were not incorporated in the 2017 ASCCP terminology. We suggest on one hand, more studies should focus on the precise extent especially the “length” of excision for different TZ types, the necessity of existence of type 2 TZ and more precise anatomic distinction between types 1 and 2 TZ. On the other hand, if evidence-based research suggests that the TZ has clinical significance, further effort to reduce heterogeneity in the classification of TZ types between individual examiners is of importance. The squamocolumnar junction is the inner margin of cervical TZ. Correctly identifying the mature columnar epithelium and then confirming the squamocolumnar junction is the key to correctly identifying the TZ.

Acetowhite epithelium is a core finding in colposcopy. Dense aceto-white epithelium had good specificity, PPV and NPV for HSIL. Major changes such as coarse mosaic, coarse punctuation, cuffed crypt openings and sharp border all had high specificity for HSIL. Two new signs, inner border sign and ridge sign also showed good diagnostic value. Compared with the major changes, the diagnostic value of minor changes signs was not satisfactory. The specificity of thin aceto-white epithelium was 59.0% and PPV 53.2%. The sensitivity of fine punctation and fine mosaic were quite low. It should be pointed out that the definition of the dense or thin aceto-white is subjective and relative, which should be combined with the type of HPV infection, the patient's age and so on. Massad et al. suggested all acetowhite lesions should be biopsied to improve sensitivity [19]. ASCCP recommended that for high-risk screening results, the biopsy of mild or translucent acetowhite changes was also necessary [28]. Actually, several signs such as punctation, mosaic, sharp border and even the new signs of both major and minor changes were highly specific and less sensitive because they occurred less frequently in cases. This reduced their diagnostic value in daily clinical practice. Therefore, we attempted to find a sign with high frequency as acetowhite changes. As we all know, the significance of Lugol’s staining was diminishing, from major changes section, minor changes section to the “nonspecific” category of the “abnormal colposcopic findings” section in 2011 Colposcopic Terminology. Our study confirmed Lugol’s staining negativity had a high sensitivity and NPV while the specificity was low. In addition, we believed Lugol’s staining was useful in delineating the boundaries of normal and abnormal tissue, identifying vaginal lesions and lesions of no obvious acetowhite changes after menopause. Lugol’s staining negativity was divided into bright and mustard yellow. We investigated the diagnostic value of bright yellow for LSIL and mustard yellow for HSIL. As a result, mustard yellow might be a valuable indicator for the diagnosis of HSIL. We believe Lugol’s staining still has certain diagnostic value in colposcopy and is the necessary procedure in colposcopic performance.

Going forward, more clinical research will be needed to improve diagnostic accuracy, and ensure that the World Health Organization’s goal of eliminating cervical cancer worldwide by 2030 is achieved. we provide some recommendations. Firstly, colposcopic terminology needs to be further refined. Signs of non-HPV 16 infection and new valuable signs need to be found. Further research is needed to confirm the existence of TZ. Secondly, colposcopy referrals should be further clarified to avoid excessive examination and insufficient examination in HPV-based screening. Thirdly, with the proper help of some biomarkers such as p16, improve the quality control of cytology, HPV detection and histopathology, so as to provide more accurate and objective clinical data for colposcopists. Fourthly, colposcopy technique is always an evolutionary process. Novel colposcopy techniques such as optical spectroscopy, computer-assisted colposcopy, electrical impedance spectroscopy, dynamic spectral imaging, confocal endomicroscopy and optical coherence tomography will need to improve and develop [29]. Last but not least, ensure the quality control of colposcopy, improve the diagnosis level of colposcopists. Adequate high-quality training and certification process for colposcopists need to be implemented. Practice makes perfect. Colposcopists should have adequate expertise and support to fulfill their role. For example, if sufficient studies confirm that the TZ is guiding for the extent of cervical conization, then we should improve the ability to accurately identify the TZ rather than denying its existence due to poor repeatability. There is no better choice than colposcopy, but there is no better choice for colposcopy than more standardized quality assurance. In this way, the benefits of changes in screening strategies can truly translate into the reduction in the incidence and mortality of cervical cancer [30].

## Conclusion

The 2011 IFCPC colposcope terminology has standardized interpretations of the colposcopic findings and improved the accuracy of colposcopy diagnosis. The aceto-white epithelium still has important diagnostic value; however, the value of a few signs is needed to be discussed and new signs are expected to be discovered. Although the significance of Lugol’s staining was diminishing, mustard yellow might be a valuable indicator for the diagnosis of HSIL.

## Data Availability

The datasets used and analyzed during the current study are available from the corresponding author on reasonable request.

## Abbreviations

IFCPC: International Federation of Cervical Pathology and Colposcopy
HPV: Human papilloma virus
HSIL: High-grade squamous intraepithelial lesion
LSIL: Low-grade squamous intraepithelial lesion
RCI: Reid Colposcopic Index
ASCCP: American Society for Colposcopy and Cervical Pathology
TZ: Transformation zone types
